# Supplementary material for: Empirical methods for controlling false positives and estimating confidence in ChIP-Seq peaks
Source: BMC Bioinformatics. 2008 Dec 5;9:523. doi: 10.1186/1471-2105-9-523 (PMC2628906; doi:10.1186/1471-2105-9-523)
Supplement: Additional File 1 — A variety of html documents from the USeq web site detailing the available applications, their best usage, output file type descriptions, command line menus, etc. [file 1471-2105-9-523-S1.zip › USeqUserGuides/JavaDoc.zip!/JavaDoc/trans/roc/class-use/ControlGene.html]

Uses of Class trans.roc.ControlGene


|  |  |  |
| --- | --- | --- |
| **Overview** \| **Package** \| **Class** \| **Use** \| **Tree** \| **Deprecated** \| **Index** \| **Help** | |  |
| PREV   NEXT | **FRAMES**    **NO FRAMES**     **All Classes** |  |


---


## **Uses of Class trans.roc.ControlGene**

| Packages that use ControlGene | |
| --- | --- |
| **trans.roc** | For manipulating the BAC dmel spike in data and generating ROC curves. |

Uses of ControlGene in trans.roc

| Methods in trans.roc that return ControlGene | |
| --- | --- |
| `static ControlGene[]` | `ControlGene.parseControlGeneFile(java.io.File file)`             parses a control gene file ex: #name chrom strand exonStarts exonEnds notes ex: NM\_001035267 chr12 + 54796640,54797239,54797532, 54796850,54797262,54797882, Z12962\_Homologue to yeast ribosomal protein L41\_H |

| Methods in trans.roc with parameters of type ControlGene | |
| --- | --- |
| `static void` | `ControlGene.makeExons(ControlGene[] cgs, int endOffSet)`             Fires makeExons on each CG within the ControlGene[]. |

---


|  |  |  |
| --- | --- | --- |
| **Overview** \| **Package** \| **Class** \| **Use** \| **Tree** \| **Deprecated** \| **Index** \| **Help** | |  |
| PREV   NEXT | **FRAMES**    **NO FRAMES**     **All Classes** |  |


---
